# Time-Lapse Analysis and Mathematical Characterization Elucidate Novel Mechanisms Underlying Muscle Morphogenesis

**DOI:** 10.1371/journal.pgen.1000219

**Published:** 2008-10-03

**Authors:** Chelsi J. Snow, Michelle Goody, Meghan W. Kelly, Emma C. Oster, Robert Jones, Andre Khalil, Clarissa A. Henry

**Affiliations:** 1 School of Biology and Ecology, University of Maine, Orono, Maine, United States of America; 2 Department of Mathematics and Statistics, University of Maine, Orono, Maine, United States of America; 3 Institute for Molecular Biophysics, The Jackson Laboratory, Bar Harbor, Maine, United States of America; University of Pennsylvania School of Medicine, United States of America

## Abstract

Skeletal muscle morphogenesis transforms short muscle precursor cells into long, multinucleate myotubes that anchor to tendons via the myotendinous junction (MTJ). In vertebrates, a great deal is known about muscle specification as well as how somitic cells, as a cohort, generate the early myotome. However, the cellular mechanisms that generate long muscle fibers from short cells and the molecular factors that limit elongation are unknown. We show that zebrafish fast muscle fiber morphogenesis consists of three discrete phases: short precursor cells, intercalation/elongation, and boundary capture/myotube formation. In the first phase, cells exhibit randomly directed protrusive activity. The second phase, intercalation/elongation, proceeds via a two-step process: protrusion extension and filling. This repetition of protrusion extension and filling continues until both the anterior and posterior ends of the muscle fiber reach the MTJ. Finally, both ends of the muscle fiber anchor to the MTJ (boundary capture) and undergo further morphogenetic changes as they adopt the stereotypical, cylindrical shape of myotubes. We find that the basement membrane protein laminin is required for efficient elongation, proper fiber orientation, and boundary capture. These early muscle defects in the absence of either *lamininβ1* or *lamininγ1* contrast with later dystrophic phenotypes in *lamininα2* mutant embryos, indicating discrete roles for different laminin chains during early muscle development. Surprisingly, genetic mosaic analysis suggests that boundary capture is a cell-autonomous phenomenon. Taken together, our results define three phases of muscle fiber morphogenesis and show that the critical second phase of elongation proceeds by a repetitive process of protrusion extension and protrusion filling. Furthermore, we show that laminin is a novel and critical molecular cue mediating fiber orientation and limiting muscle cell length.

## Author Summary

Despite the importance of muscle fiber development and tendon attachment, this process is incompletely understood in vertebrates. One critical step is muscle fiber elongation; muscle precursor cells are short and subsequent elongation/fusion generates long, multinucleate muscle fibers. Using a vertebrate model organism, the zebrafish, we find that single round myoblasts elongate to span the entire width of the myotome prior to fusion. Using rigorous and objective mathematical characterization techniques, we can further divide muscle development into three stages: short precursor cells, intercalation/elongation, and boundary capture/myotube formation. The second phase, elongation, occurs via a two-step mechanism of protrusion extension and filling. Myotube formation involves boundary capture, where the ends of muscle fibers anchor themselves to the myotome boundary and stop elongating. We show that the protein laminin is required for boundary capture, normal fiber length, and proper fiber orientation. Genetic mosaic experiments in laminin-deficient embryos reveal that boundary capture is a cell autonomous phenomenon. Wild-type (normal) cells capture the boundary appropriately and stop elongating in laminin-deficient embryos. Although adhesion to laminin has been implicated in muscular dystrophies where the attachment between muscle cells and tendons fails, no early developmental requirements for laminin in fast muscle morphogenesis have been shown until now.

## Introduction

Muscle specification and morphogenesis during early development are critical for normal muscle physiology. In vertebrates, most of the musculature is derived from somites [1]–[3]. Somites are segmentally reiterated structures delineated by somite boundaries. As development proceeds, a portion of the somite gives rise to skeletal muscle fibers that comprise the myotome. The terminal ends of myotomal muscle fibers attach to somite boundaries, which then become myotome boundaries. In teleost fishes, myotome boundaries give rise to the myotendinous junction (MTJ) [4].

Myotome development is perhaps best understood in amniotes. Myogenesis in amniotes begins when muscle precursor cells translocate from the overlying dermomyotome to the myotome [3]. The first myocytes to translocate come from the dorsomedial lip, but later in development myocytes translocate from all dermomyotome borders as well as the central region [5]–[7]. Time-lapse analysis in the chick embryo has shown that spatial domains of the somite differ in cell behaviors that generate the primary myotome [8]. The above studies have elucidated cell movements that generate the myotome. However, although it is known that short, mononucleate, muscle precursor cells generate long, functional multinucleate muscle fibers, it is not known how this occurs. Interestingly, the early zebrafish and chick myotomes have been described as containing mononucleate muscle fibers [8],[9]. Our intent in undertaking this study was to utilize the advantages of the zebrafish system to shed light on early muscle development in a vertebrate model.

Elucidation of the cellular mechanisms that underlie muscle fiber development and tendon attachment is critical for a comprehensive understanding of muscle development. Towards this end, muscle morphogenesis has been studied in different model systems. C2C12 myoblasts in culture elongate slightly prior to differentiation, align with each other, and fuse to generate a multinucleate myotube [10]. We have called this scenario elliptical growth. In grasshopper embryos, the first muscle cells elongate between attachment sites prior to fusion. These cells extend many processes in multiple directions while elongating [11]. We have termed this scenario branching. Elegant studies in *Drosophila* have shown that muscle morphogenesis occurs when myoblasts fuse to generate long, multinucleate myotubes and identified a number of proteins required for myoblast fusion [12],[13]. Extremely exciting recent studies have shown that there is some conservation of molecular mechanisms that mediate muscle cell fusion between *Drosophila* and zebrafish [14],[15]. Interestingly, however, the primary myotome in chick and zebrafish is mononucleate [8],[9]. This suggests that myoblast fusion does not mediate the earliest stages of muscle morphogenesis in vertebrates, but occurs after initial muscle fiber elongation. These distinct mechanisms of morphogenesis in different systems highlight the fact that a mechanistic study of muscle fiber morphogenesis in vertebrates has not yet been undertaken. Identification of discrete morphogenetic steps that mediate muscle fiber morphogenesis in vertebrates is necessary to provide a framework for future molecular analyses.

Adhesion of muscle fibers to the basement membrane is critical for muscle function. The basement membrane attaches muscle fibers to connective tissue that then attaches to the skeletal system; this attachment is critical for force transduction from muscle to bone. One major component of the basement membrane is laminin. Laminin is a heterotrimeric protein composed of α, β and γ subunits that generate at least 15 different isoforms [16]. The importance of laminins in muscle physiology is evidenced by the fact that mutations in *lamα2* result in muscular dystrophies [17]–[20]. Recent work has shown that muscle fibers in zebrafish mutant for *lamα2* elongate and attach to the MTJ, but at 48 hours post fertilization (hpf) fibers detach before death, providing novel insight into roles for *lamα2* in muscle disease [21].

Significantly less is known about spatiotemporal mechanisms of basement membrane assembly during early skeletal muscle development and whether adhesion to the basement membrane contributes to morphogenesis. Recent data suggest that the laminin receptor Integrin α6β1 is necessary for both basement membrane assembly and normal expression of myogenic regulatory factors in cultured mouse explants [22]. However, Integrin α6β1 binds to multiple laminins with distinct affinities [23] and roles for individual laminin chains during early muscle development *in vivo* have not been identified. In order to determine whether the basement membrane is critical during early development for muscle fiber elongation and attachment, it is first necessary to understand the cellular basis of muscle fiber elongation and attachment.

The relative simplicity of zebrafish skeletal muscle, where slow and fast-twitch fibers are spatially segregated, makes it an ideal model system to study muscle cell elongation and MTJ morphogenesis. Morphogenesis of the somite boundary into the MTJ involves three stages: initial epithelial somite boundary formation, transition and myotome boundary/MTJ formation [24]. Transition encompasses the lateral displacement of slow-twitch muscle fibers and the subsequent elongation and differentiation of fast-twitch fibers [25]–[28]. The initial myotome forms by 26 hpf and contains long muscle fibers attached to the myotome boundary/MTJ. At this point, the extracellular matrix (ECM) proteins Fibronectin, laminin and Periostin concentrate at the MTJ [29]–[31]. Morpholino-mediated inhibition of Periostin disrupts MTJ formation [29],[32],[33], but discrete and mechanistic requirements for other ECM proteins and their receptors are not known. In addition, the precise mechanism by which elongating muscle fibers attach to the MTJ and cease elongation has not been elucidated.

The purpose of this study was to rigorously and quantitatively characterize, for the first time in vertebrate embryos, the cellular events that generate long myotubes from initially short muscle precursor cells. We focused on fast-twitch fiber morphogenesis in zebrafish embryos. Our goal was to develop methods with which discrete functions for proteins involved in muscle morphogenesis could be identified. Towards this goal, we utilized time-lapse analysis, genetic mosaic analysis, and three different mathematical tools including a powerful wavelet-based image analysis formalism to provide novel insight into cellular and molecular mechanisms that underlie muscle fiber elongation and subsequent attachment to the nascent MTJ.

## Results

### Three Phases of Fast-Twitch Muscle Cell Elongation

**Figure 1:**
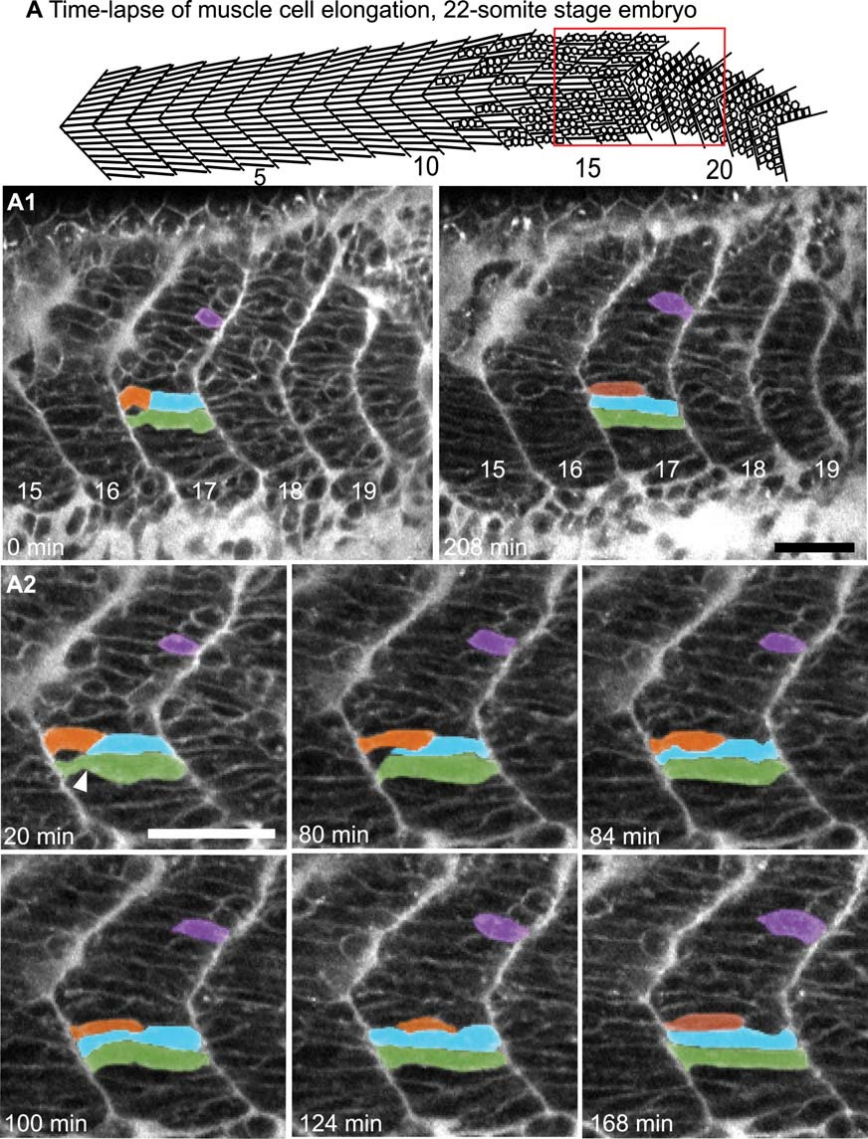
Myoblasts Intercalate between Each Other as They Elongate. (See also Movies S1 and S2.) A) Cartoon depicts the anterior to posterior progression of myofiber elongation in a 22 somite embryo. A1–A2) Confocal time-lapse sequence showing fast muscle cell elongation in a single focal plane of a zebrafish embryo vitally labeled with BODIPY-Ceramide. Anterior left, dorsal top, somite number denoted, time elapsed indicated on panels. The colored cells were pseudocolored to facilitate visualization. By 80 min, the blue cell is beginning to intercalate, intercalation is complete by 84 min. During this time, the orange and purple cells are elongating. The green cell transits from a long, but irregularly shaped cell (white arrowhead indicates a groove at 20 min) into a rod-shaped myotube by 124 min. Scale bars: 50 µm.

Although the elongation of somitic cells is critical for actin-mediated contractility that underlies muscle function, the cellular and molecular basis of elongation in vertebrates is not well understood. An understanding of how muscle cells elongate is critical in order to determine mechanistic roles for genes required in elongation. We used time-lapse microscopy of zebrafish embryos labeled with BODIPY-Ceramide to outline cells. This type of time-lapse analysis, where all cells are labeled, provides an initial framework with which to focus further investigation into fast-twitch fiber morphogenesis. Fast-twitch muscle cells can be identified because, in contrast to slow-twitch muscle fibers, they are not migrating medially-laterally [25]. The transition from a somite to a myotome is a dynamic process (Figure 1, Movie S1) with at least three phases. The first phase is short muscle precursor cells. Second, muscle fibers elongate by extending narrow protrusions to intercalate between other cells (Figure 1 A2 at 80 min, blue pseudocolored cell). Elongation ends when cells adhere to the anterior and posterior boundaries. The third phase is myotube formation. Recently elongated cells are long, but irregularly shaped (Figure 1 A1 green cell at 0 min, A2 blue cell at 84–168 min, Movie S1). During myotube formation, long cells with grooves continue to change shape until they form a more uniformly shaped tube without grooves (Figure 1 A1 green cell at 208 min). An additional time-lapse is shown in Movie S2.

### Phase 1: Short Muscle Precursor Cells

**Figure 2:**
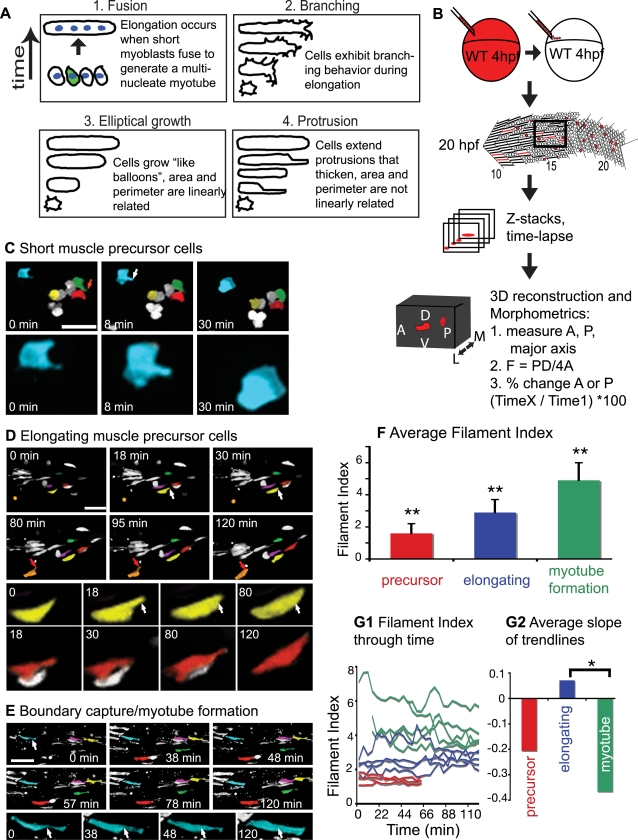
Three Phases of Muscle Morphogenesis: Short Muscle Precursor Cells, Intercalation/Elongation, and Boundary Capture/Myotube Formation. (See also Movie S3.) Projections of ApoTome micrographs are shown, side views, anterior left, dorsal top. Cells were pseudocolored to facilitate visualization. A) Cartoon depicting possible scenarios for elongation of myofibers. B) Cartoon depiction of the methods used. Dextran filled WT cells (red) were transplanted into an unlabeled embryo at the blastula stage, time-lapse data was collected at 20 hpf, then Z-stacks were three dimensionally projected for morphometric analysis. C) Short muscle precursor cells do not undergo large-scale shape changes. A 21 somite-stage embryo, approximate location of somite 15 at left. The blue cell extends a filopodia-like protrusion (8 min, white arrow) that is then retracted (30 min, the blue cell is enlarged in the bottom panels). The protrusion in the green cell (red arrow at 0 min) is also retracted by 30 min. D) Elongating muscle precursor cells extend protrusions along their major axis as they elongate. A 22 somite-stage embryo, somite 18 at left. The yellow cell extends a long, thin protrusion (white arrow) at 18 min that increases in thickness, resulting in a longer cell. The orange cell extends a protrusion (red arrow) at 80 min that becomes thicker by 120 min. E) Myotube formation involves the transition from an irregularly shaped cell to a more homogenously shaped tube. At 0 min, the blue cell with a white arrow is not yet tube-shaped, i.e. part of the cell is significantly narrower than the other parts (white arrow). Over time, the narrow portion thickens, eventually generating a long tube-shaped myotube (120 min, white arrows in bottom enlarged panels are in the same location in all panels). F) The filament index is significantly different between the three phases (**, p<0.01). G1) The filament index of the three phases through time. G2) Average slopes of linear trendlines from data in G1 (*, p<0.05).

A three-dimensional quantification of cell morphology is critical to distinguish between scenarios of muscle cell elongation (Figure 2A). We transplanted dextran-filled cells into unlabeled host embryos and three-dimensionally reconstructed the behavior of labeled cells through time (Figure 2B). For each time point, the z-series was three-dimensionally projected and the area, perimeter, and major axis were measured. Thus two-dimensional parameters (area, perimeter, and major axis) were obtained from three-dimensional projections of cells. The analysis of labeled cells in an unlabeled field of cells allows unambiguous determination of cellular shape dynamics and quantification of morphometric parameters. We analyzed cell behaviors in two ways: (1) analysis of the filament index and (2) analysis of the relative dynamics of area and perimeter changes through time. As shown below, this approach supports and extends what was observed in BODIPY-Ceramide labeled embryos.

The filament index is an excellent mathematical parameter that describes cell morphology. The filament index is a measure that quantifies the departure of a shape from a circle (see methods). A circle has a filament index of 1 and a higher filament index indicates a larger departure from a circular shape. Short muscle precursor cells have a low filament index (FI) indicating that their morphology is close to a circle (Figure 2F, G1, FI = 1.6±0.6, Table 1).

**Table 1:** Morphometric analysis of muscle cells.

Phase	Subcategory	N	Average slope±SE	Major Axis vs. Perimeter R^2^	Filament Index±SD
**Short precursor**	WT live	12		0.57	1.6±0.6
	WT fixed	31		0.59	1.4±0.2
	Laminin-deficient fixed	8		0.92	1.4±0.3
**Elongating precursor**	WT live	6		0.9	2.9±0.8
**WT live**	% change in area	6	2.7±0.8		
**WT live**	% change in perimeter	6	1.8±0.8		
	WT fixed	255		0.90	
	Laminin-deficient fixed	166		0.89	3.0±1.5
**Forming myotube**	WT live	6		0.31	4.9±1.1
**WT live**	% change in area	6	1.5±0.5		
**WT live**	% change in perimeter	6	0.2±0.1		
	WT fixed	13		0.16	6.0±0.9
	Laminin-deficient fixed	11		0.9	5.1±2.2

Short muscle precursor cells extend and retract very short (<2 µm) filopodia-like protrusions in all directions (Figure 2C, Movie S3). Small changes in the area, perimeter, and length of muscle precursor cells reflect the dynamic shape changes of precursor cells (not shown). However, their overall shape and size remains consistent.

### Phase 2: Elongating Fast-Twitch Muscle Precursor Cells

Elongating cells lengthen towards their attachment site, the MTJ. Elongating cells have a higher filament index than short precursor cells (Figure 2F, 2.9±0.8, Table 1). The filament indices of elongating cells increase slightly through time (Figure 2G1, G2), reflecting their departure from a circular shape.

One purpose of this experiment was to distinguish between possible scenarios of muscle fiber elongation summarized in Figure 2A. The difference between the fusion and remaining scenarios is the timing of fusion relative to elongation. In the fusion scenario, fusion of short myoblasts is the major morphogenetic event that drives fiber elongation. Fusion of multiple short cells generates a long, multinucleate myotube in one step as in *Drosophila* [12]. In the remaining scenarios, cells elongate prior to fusion. We analyzed nuclear content of elongating and recently elongated cells and found that mononucleate fast-twitch cells elongate to the MTJ prior to fusion (Figure 4F–H, n = 108 cells). Thus, the first fast-twitch fibers in zebrafish do not fuse prior to elongation.

The remaining scenarios are branching, elliptical growth, and protrusion (Figure 2A). The difference between the branching scenario and the elliptical growth/protrusion scenarios is the amount, size, and direction of protrusive activity. In grasshopper, the first muscle cells to elongate have extensive protrusions in many different directions [11]. This is depicted in the branching scenario (Figure 2A). In contrast, the elliptical growth and protrusion scenarios depict cells that elongate in a fixed direction. Both time-lapse analysis and analysis of cell morphology in fixed embryos indicate that fast-twitch fibers in zebrafish embryos elongate in a fixed direction and do not exhibit a branching morphology with multiple protrusions extended in different directions (Figures 2, 4). Rather, fast-twitch cells extend long (>4 µm) protrusions along their long axis (Figure 2D, Movie S3). These results indicate that the branching scenario does not apply to initial fast-twitch muscle morphogenesis in zebrafish.

**Figure 3:**
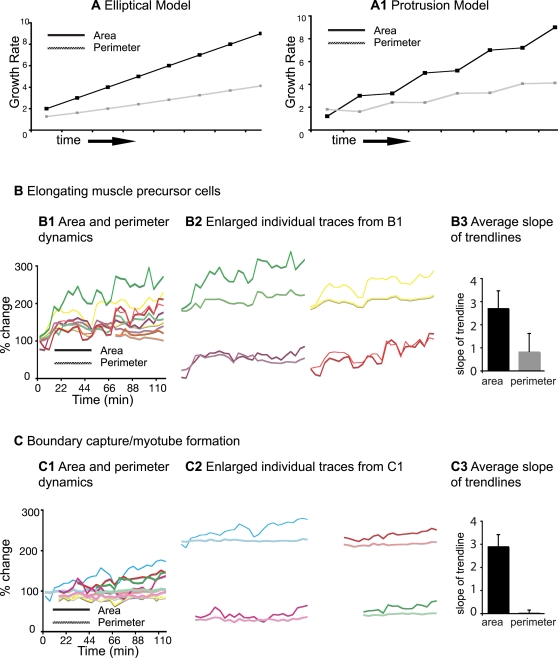
Mathematical Analysis of Area and Perimeter Dynamics. A) Geometric modeling of relative changes in area and perimeter that would result if cells elongated as ellipses, as if they were filling like balloons. Both area and perimeter would increase linearly. A1) Geometric modeling of relative changes in area and perimeter that would result if cells elongated via a two-step process: protrusion extension and filling. Both area and perimeter would increase incrementally. B) Area and perimeter dynamics during the elongation phase. B1) Percent change of area (solid line) and perimeter (dotted line) through time. Enlarged traces are shown in B2. B3) Average slope of linear trendlines. The area increased more than the perimeter in all cells. C) Area and perimeter dynamics during the myotube formation phase. The growth rate of area and perimeter are different in the myotube formation phase than the elongation phase. As the cell transitions from being irregularly shaped to a more homogenously shaped myotube, the increase in area represents the filling in of an initially narrow aspect of the cell. Filling in does not dramatically increase the perimeter, but does result in an increase in area.

The two remaining scenarios differ in the nature of elongation. The elliptical growth scenario depicts elongation as a continuous process reminiscent of a balloon filling. The protrusion scenario suggests that elongation is incremental and proceeds via a 2-step mechanism: protrusion extension and protrusion thickening. Time-lapse analysis suggests that fast-twitch cells extend protrusions that subsequently thicken (Figures 1, 2). Geometrical models were developed and used to determine how area and perimeter would change through time if cells were elongating via the two different scenarios. The major difference between the two models is the nature of dynamic changes in area and perimeter during elongation (see methods for details). Area and perimeter increase linearly in the elliptical growth model (Figure 3A) and incrementally in the protrusion model (Figure 3 A1). The incremental nature of growth in the protrusion model is because the perimeter increases slightly more when the protrusion extends, but the area increases slightly more when the protrusion thickens (Figure 3 A1). Therefore the difference between the two models is how area and perimeter values change as the cell grows: linear changes occur in the elliptical growth model and incremental changes in the protrusion model. In all cells examined, the rate of area increase is higher than the rate of perimeter increase as is predicted by both models (Figure 3 B3). However, area and perimeter increase incrementally during fast-twitch muscle cell elongation (Figure 3 B1, B2). Thus, analysis of area and perimeter dynamics supports the two-step intercalation model.

### Phase 3: Boundary Capture/Myotube Formation

Boundary capture occurs when elongating muscle cells reach myotome boundaries and stop extending. The filament index of cells in the boundary capture/myotube formation phase is significantly higher than the preceding two phases (Figure 2F, Table 1). Their filament indices decrease slightly through time (Figure 2 G1, G2). This decrease reflects the fact that a rod-shaped cell has a similar perimeter and length, but larger area than a long, irregularly shaped cell.

Recently elongated cells can be irregularly shaped and of varying diameters (Figure 2E). The thinner portions of the cell then thicken until the entire cell consists of a more uniform diameter (Figure 2E, Movie S3). Note that changes in area and perimeter of cells in the myotube formation phase are distinct: in this phase the area increases much more than the perimeter (Figure 3C, compare C3 to B3). The increase in area without a substantial increase in perimeter reflects the adoption of a more tube-shaped, regular morphology in the myotube formation phase.

Taken together, the time-lapse data along with two different quantitative analyses (area and perimeter dynamics through time and the filament index) indicates that there are three discrete morphogenetic phases that generate the first fast-twitch muscle fibers: short muscle precursor, intercalation/elongation and boundary capture/myotube formation.

### Analysis of Fast-Twitch Muscle Cells in Fixed Embryos

**Figure 4:**
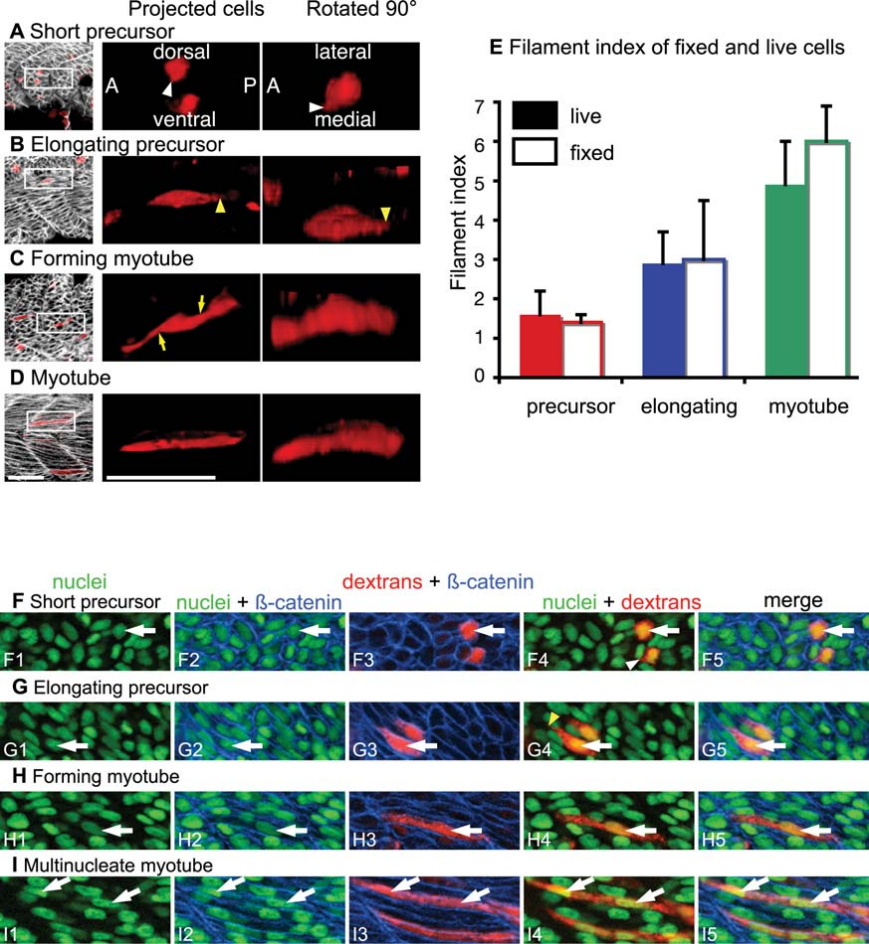
Mathematical Characterization of Fixed Cells Supports Time-Lapse Analysis of Live Cells. (See also Movie S4.) ApoTome images, side views, anterior left, dorsal top, 22 somite-stage fixed embryos. A–D) Dextran-labeled cells (red), β-catenin staining outlines cells (white). Rotated 90° projections were stretched≈3 fold in the Z dimension due to the relative thinness of the tissue. Scale bars: 50 µm. A) Short muscle precursor cells are short and extend small protrusions (white arrowheads). B) Long protrusions extended along the major axis of the cell are observed (yellow arrowheads). C) Irregularly shaped cells that span the entire width of the myotome are observed (yellow arrows). D) Long regularly shaped myotube. E) Graph showing that the filament indexes for live and fixed cells are similar. F–H) 22 somite-stage fixed embryo, nuclei are stained with Sytox green, dextran-labeled cells are red, β-catenin in blue outlines cells. Although the nuclear content of cells was analyzed in all focal planes, only one focal plane is shown for clarity. White arrows point to nuclei. F) Precursor cells are short and have one nucleus (white arrowhead in F4 shows short protrusion). G) Cells that were elongating when fixed were never observed to contain more than 1 nucleus (yellow arrowhead shows a long protrusion). H) An elongated cell with 1 nucleus. I) Multinucleate myotube.

Morphometric analysis of fixed cells corroborates the time-lapse data. Myotome formation proceeds in an anterior-posterior progression. Thus, this approach allows analysis of muscle cells in various stages of elongation within the same embryo. As observed in live embryos, fixed muscle precursor cells are short (<5 µm) and have short protrusions (Figure 4A, F, white arrowheads). Protrusions in fixed cells are also observed to extend in all directions (Figure 4A). The filament index of live precursor cells and fixed precursor cells is similar (Figure 4E live cells 1.6±0.6, fixed cells 1.4±0.2). The correlation of the length with perimeter is also similar to live cells (Table 1).

Both the qualitative appearance and the morphometric properties of fixed cells presumed to have been elongating (those between 5 µm and 40 µm in fixed embryos) are similar to live elongating cells. The major axis is very strongly correlated with perimeter and area in both populations (Table 1). Similar to live cells, long narrow protrusions are only observed along the major axis (Figure 4B, G, yellow arrowheads, Movie S4). We also analyzed the nuclear content of dextran-filled cells during elongation. A z-series was taken and cells were examined in three dimensions. No elongating cells contained more than one nucleus (Figure 4G, n>100 cells).

Cells that were fully elongated but irregularly shaped were presumed to be in the boundary capture/myotube formation phase (Figure 4C is a three-dimensional projection of an irregularly shaped but elongated cell). The filament index of these cells was also similar to live cells (Figure 4E). Fast-twitch cells in this phase were mononucleate (Figure 4 H shows one focal plane of a mononucleate cell that is elongated but irregularly shaped when examined in three dimensions, Movie S4, n = 108). Interestingly, all muscle cells that contained multiple nuclei exhibited a stereotypical tubular shape (n = 57, see Figure 4I). These data suggest the intriguing possibility that the transition from an irregularly shaped long cell to a rod-shaped myotube may involve fusion. Our use of dextran-labeled cells in a field of unlabeled cells clearly highlights the morphological complexity of elongating fast-twitch muscle cells and indicates that it is not possible to unambiguously identify multinucleate cells utilizing a nuclear marker as well as a marker that denotes all cells (such as phalloidin). Thus, we do not know the exact timing of muscle cell fusion or whether fusion contributes to the morphogenesis of irregularly shaped long fibers into regularly shaped, cylindrical myotubes. However, it is evident that the first fast-twitch muscle cells do not fuse in order to elongate.

The filament index of fixed muscle cells, as in live cells, is significantly different between each phase (data not shown, two-tailed t-test, p<0.01 for all comparisons). These data support the time-lapse analyses and provide new tools for analysis of morphogenetic defects in various mutant/morphant embryos.

### Quantification of Anisotropy Indicates that Each Phase of Muscle Fiber Morphogenesis Is Accompanied by a Significant Increase in Ordered Structure

**Figure 5:**
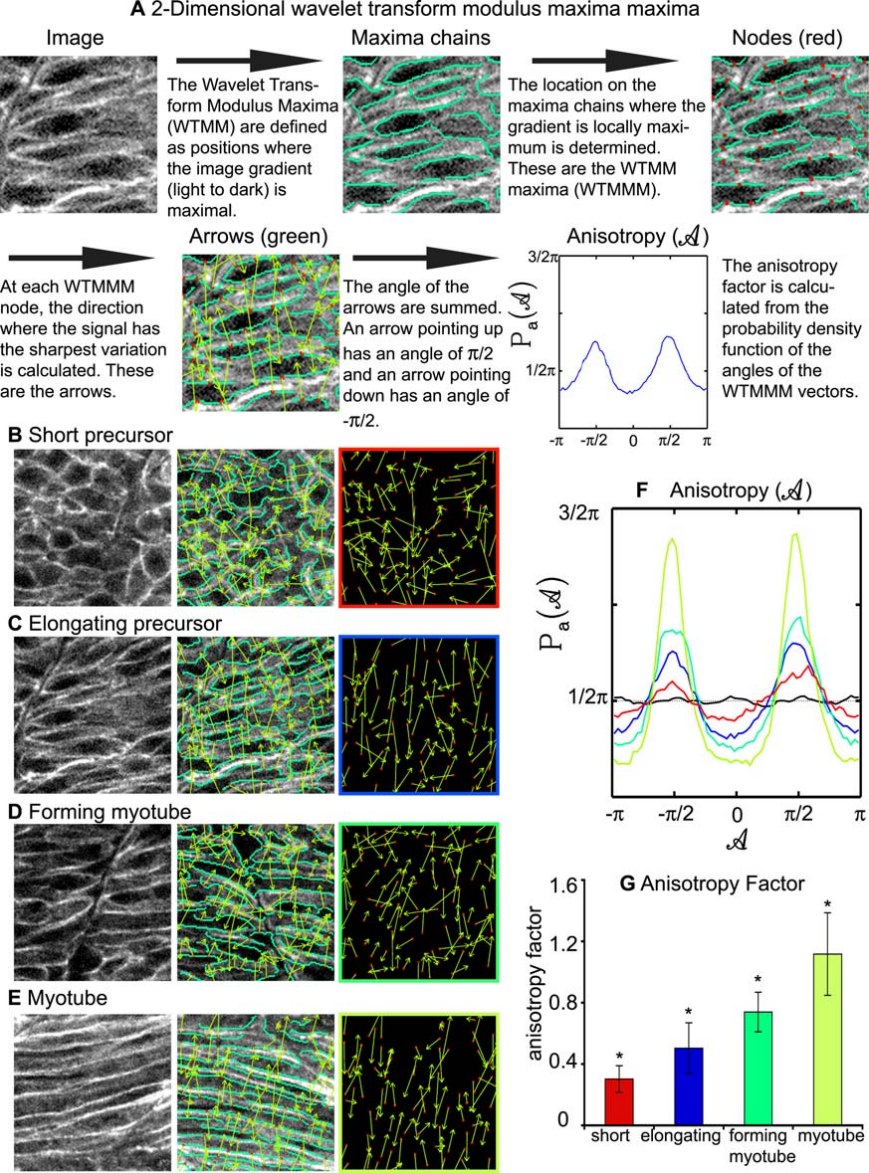
The 2D WTMM Method Is Used to Quantify Cellular Structure within a Lattice, and Indicates that Cellular Organization Increases during Muscle Morphogenesis. A) Description of how the 2D WTMM formalism quantifies structure. The starting image is of elongating muscle precursor cells stained for β-catenin to outline cells. B) Short muscle precursor cells have almost all WTMMM vector arrows pointing in random directions, indicating that there is only a small departure from isotropy (isotropy means randomly structured). C) Organization increases as muscle cells begin to elongate. Note more green arrows pointing either up or down in C than B. D) Organization continues to increase during the myotube formation phase. E) Organization is readily apparent when myotubes have formed. Note that most of the green arrows are pointing either up or down indicating high levels of organization. F) Averaged *P_a_*(*A*) for one particular size scale (a∼7 µm) for the myotube stage (lime green curve), the forming myotube stage (dark green curve), elongating precursor stage (blue curve), short precursor (red curve) as well as for the isotropic fBm surfaces analyzed for calibration purposes (black curve fluctuating around π/2). Also shown is the flat 1/2π curve that would be obtained for a purely theoretical isotropic process (flat pointed line at 1/2π). G) The anisotropy factor *F_a_* was averaged over all size scales analyzed. An indicator of organized structure, it shows significantly distinct values for all stages of developing muscle cells.

The identification of discrete, mathematically distinct phases provides a paradigm by which muscle morphogenesis in mutant embryos can be assessed. The above data also indicate that the morphology of fixed cells is not significantly different than live cells. However, although obtaining single labeled cells within a field of unlabeled cells in fixed embryos is easier than time-lapse analysis, it is not feasible in all model systems. We thus looked for a different mathematical tool to quantify cellular organization. Ideally such a tool would allow objective quantification of cellular structure with an easier experimental preparation such as staining with phalloidin to outline all cells. Therefore, we adapted and applied the 2D Wavelet-Transform Modulus Maxima (WTMM) method [34],[35]. This method can be used to quantify the amount of structure, or order, of objects that do not necessarily have a well defined boundary. We used this approach to quantify the structural organization of cellular lattices during muscle fiber elongation. The WTMM analysis filters an image with the gradient of a smoothing function (i.e. a wavelet) at a given size scale. Places within the image where the intensity variation is maximal are given by the wavelet-transform modulus maxima (i.e. the WTMM). Next, the positions of maximal intensity variation along these maxima chains are identified. These are the WTMM maxima, or WTMMM. At these nodes, the direction where the signal has the sharpest variation is calculated. An arrow that points upward has an angle of π/2 and an arrow that points down has an angle of −π/2. The anisotropy factor *F_a_* is then calculated from the probability density function, *P_a_*(*A*), of the angles *A* of the WTMMM vectors. *F_a_* is defined in such a way that randomness, isotropy, has a value of *F_a_* = 0. Any value of *F_a_*>0 quantifies the extent of departure from isotropy. A randomly structured cell lattice has arrows pointing in all directions and a low anisotropy factor. The arrows point in all directions because the direction of maximal intensity variation is random. A more organized cell lattice will have more arrows pointing in the same direction and a stronger anisotropic signature. More arrows will point in the same direction in an ordered cell lattice because the direction of maximal intensity variation will be the same between multiple cells. Thus, this formalism objectively provides a quantitative assessment of morphological structure. A step-by-step explanatory diagram is presented in Figure 5.

The WTMM analysis was applied for all size scales between *a*∼4 and *a*∼13 µm (see methods for details on staining and image preparation). Short muscle precursor cells have a low anisotropy factor indicating that there is only a small departure from isotropy. Organization increases throughout muscle elongation. This increase in organization through time is visible as the increase in the proportion of arrows pointing in the same directions (Compare Figure 5B to 5E). The averaged probability density functions *P_a_*(*A*) over all size scales *a* are shown in Figure 5F and the resulting averaged anisotropy factors *F_a_* are shown in Figure 5G. Each phase of muscle development has a significantly higher anisotropy factor indicating increasing cellular organization through time (importantly, statistical significance is maintained when only single size scales are used).

Taken together, all the methods used (time-lapse analysis, area/perimeter dynamics, filament index, and 2D WTMM) show that there are discrete phases of fast muscle morphogenesis. Furthermore, the fact that the 2D WTMM analysis supports the other morphometric analyses used indicates that this is an exceedingly valuable tool that can objectively quantify how ordered/structured a field of cells is without having to isolate or segment individual cells.

### *lamβ1* and *lamγ1* Are Required for Normal Fast Muscle Cell Orientation

Thorough knowledge of the cellular mechanisms underlying muscle fiber elongation provides a framework for elucidating the molecular basis of muscle cell elongation. We asked if a prominent basement membrane protein, laminin, is required for muscle morphogenesis. It is known that a laminin receptor, Integrin α6β1, is required for normal myofiber development in cultured mouse explants [22] but the relevant laminin ligands are unknown.

**Figure 6:**
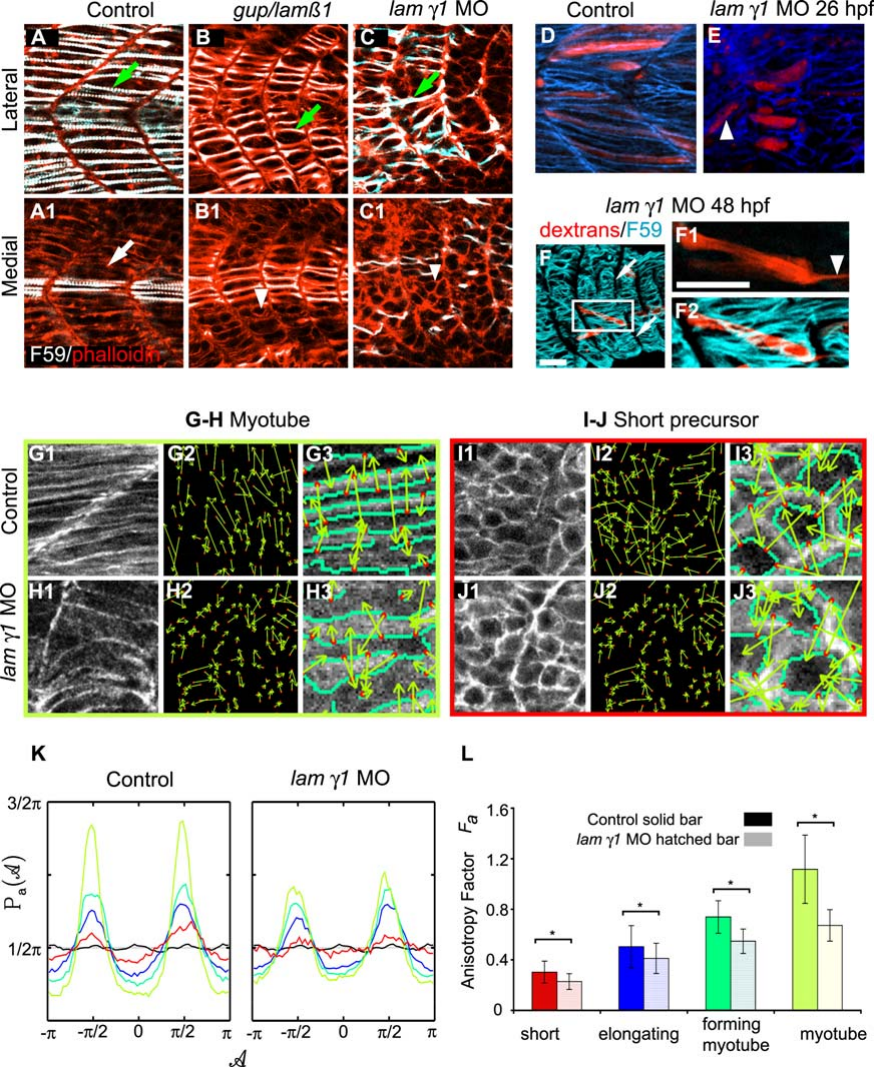
*lamininβ1* and *γ1* are Required for Normal Fast Muscle Cell Elongation. Panels A–C are confocal images and D–J are ApoTome micrographs. Panels A–C are side views, anterior left, dorsal top of 18 somite-stage embryos stained with F59 (white) to denote slow-twitch muscle and phalloidin (red) to outline fast muscle cells. Panels labeled 1 are lateral sections from a z-series and panels labeled 2 are medial sections from the same z-series. A) In WT embryos, fast-twitch muscle cells medial (A1, white arrow) to migrating slow-twitch fibers (A, green arrow) have elongated. B) Although some slow-twitch fibers do migrate in *gup/lamβ1* mutant embryos, not all fast muscle precursor cells have elongated (B1 white arrowhead: short cell, B green arrow: slow-twitch muscle fiber that has migrated laterally). C) Not all fast muscle precursor cells medial to migrating slow fibers have elongated in *lamγ1* morphant embryos (C1 white arrowhead: short cell, C green arrow: slow-twitch muscle fiber that has migrated laterally). Panels D–E are projected views of dextran filled cells (red) and β-catenin that outlines cells (blue). D) Elongated fibers in a WT embryo, note the organized, parallel array of fibers. E) Elongated fibers in a *lamγ1*-deficient embryo, white arrowhead denotes a fiber that is not parallel. F–F2) A dextran-filled cell in a *lamγ1* morphant embryo extends a thin protrusion across the MTJ. White arrows denote the MTJ, white arrowhead denotes thin protrusion extending across the MTJ. F59 denotes slow muscle in blue and dextrans are red. Panel F1 is a single focal plane from a z-series, panels F and F2 are projections. Scale bars F: 50 µm, F1: 20 µm. G–H) Cells in the myotube phase are less organized in *lamγ1* morphant embryos than in control embryos as shown by more randomly oriented WTMMM vector arrows. Panels numbered 3 are higher magnification views. I–J) Although differences in cellular structure are not obvious to the eye (compare I1 and J1), *lamγ1* short precursor cells are less organized than control cells as shown by more randomly oriented WTMMM vector arrows. Panels numbered 3 are higher magnification views. K: The WTMMM vector angle pdfs are displayed for all stages (color coded per panel L), the isotropic fBm surfaces (black curve fluctuating around π/2), and the flat 1/2π curve that would be obtained for a purely theoretical isotropic process (flat pointed line at 1/2π). Note the stronger (higher) peaks in control embryos. L) The anisotropy factor of muscle cells in laminin-deficient embryos is significantly lower than in control embryos at all four stages of muscle morphogenesis (p<0.01). These results indicate that even though differences in organization as far back as the precursor stage are not obvious visually, they are unequivocally more disorganized than in controls when the anisotropic value is determined.

We find that muscle cell elongation in *lamβ1* and *γ1* mutants and morphants is delayed. In zebrafish, slow-twitch fibers migrate laterally and trigger fast muscle cell elongation [28]. Thus, slow fiber location is an excellent marker for assaying fast muscle cell elongation: fast cells medial to slow fibers should be fully elongated. Although slow muscle fiber migration is disrupted in *lamβ1* and *γ1*-deficient embryos (Figure S1), some slow fibers migrate laterally. However, fast muscle cells medial to migrating slow fibers are short in *lamβ1* or *lamγ1* mutants and morphants (Figure 6B, C, and data not shown, n = 6 *grumpy/lamβ1* mutant embryos, 16 *lamβ1* morphant embryos, 5 *wi390/lamγ1* mutant embryos and 10 *lamγ1* morphant embryos).

Fast-twitch muscle cells belatedly elongate in *lamβ1* and *γ1*-deficient embryos and the filament index of cells in all three phases is similar to control embryos (Table 1). However, fast muscle cells frequently appear misoriented in *lamβ1* and *γ1*-deficient embryos (Figure 6E, note the abnormal angle of cells that are not aligning in a parallel array, data not shown). Application of the 2D WTMM method indicates that myotubes in *lamγ1*-deficient embryos are significantly more disorganized than in control embryos. Elongated myotubes in control embryos form an organized array as indicated by the strong polarization of the yellow arrows (Figure 6 G3). The arrows tend to point either up or down resulting in high peaks at π/2 and −π/2 (Figure 6K lime green line) and a higher anisotropy factor (Figure 6L). In contrast, arrows in *lamγ1*-deficient embryos are far less polarized (compare Figure 6 H3 to G3). The peaks at π/2 and −π/2 are lower than in wild-type embryos (Figure 6K lime green line) and the anisotropy factor is significantly lower (Figure 6L). Thus, application of the 2D WTMM formalism quantitatively supports the qualitative perception that muscle fibers are disorganized in *laminin*-deficient embryos.

The next question that follows is *when* does the anisotropic signature in *laminin*-deficient embryos become different from wild-type embryos? No overt morphological differences between control and *laminin*-deficient cells in the short precursor phase are visible to the eye (Figure 6I, J). However, there is a slight but significant difference between the anisotropy factors (Figure 6L). The difference between anisotropy factors increases at every phase of muscle morphogenesis. These data indicate that *laminin* is required for cellular organization as early as the short precursor phase. Thus, subsequent myotube disorganization may reflect both early and late requirements for laminin during muscle morphogenesis.

### Laminin Is a Molecular Cue that Stops Fiber Elongation

**Figure 7:**
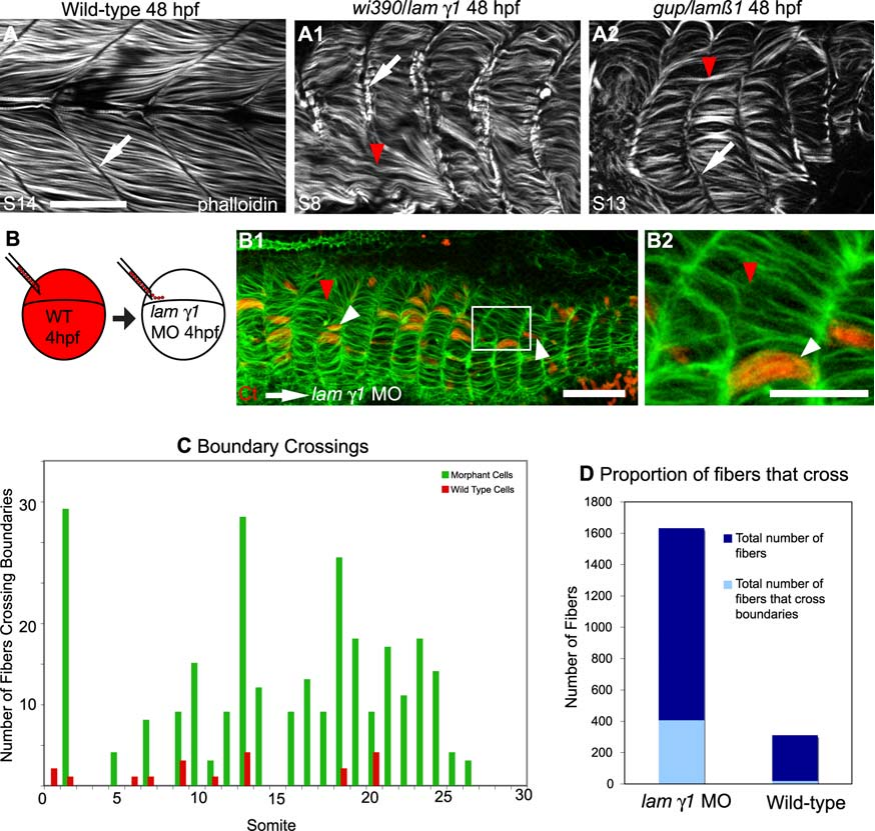
Laminin Plays a Role in Boundary Capture of Elongating Muscle Fibers. ApoTome micrographs, side views, anterior left, dorsal top of 48 hpf embryos. A–A2) MTJ boundaries are sometimes crossed in *lamβ1* and *lamγ1*-deficient embryos. The MTJ in WT embryos is visible as the dark line of no phalloidin staining in between myotomes (A, white arrow). In both *lamβ1* and *γ1* mutants, MTJs are observed (A1–A2, white arrows), but sometimes a portion of an MTJ is crossed by a muscle fiber (red arrowheads, A1–A2). Scale bar: 50 µm. B–B2) Cell autonomous rescue of boundary crossing by control cells in *lamγ1* morphant embryos. White box in B1 indicates the higher magnification view in B2. Transplanted control cells do not cross the MTJ boundary (white arrowheads, only 19/311 transplanted control cells crossed MTJ boundaries in *lamγ1* morphant embryos compared to 402/1631 morphant cells). The red arrowhead indicates morphant cells that cross boundaries. Scale bars: 20 µm. C–D) Graphs showing boundary crossing by control cells and morphant cells.

It has been proposed that muscle fibers elongate until they reach a small patch of ECM that functions to capture elongating muscle cells and prevent them from extending into the next myotome [24]. However, it is not known which of the many ECM components of the MTJ are required or if multiple proteins are required. We find that both *lamβ1* and *lamγ1* play a role in MTJ morphogenesis. Some fast muscle cells in *lamβ1* and *lamγ1* mutants and morphants do not stop elongating at the MTJ (Figure 6 F1 white arrowhead, note that the muscle cell extends a long, thin protrusion across the boundary). The MTJ is visible in 48 hpf wild-type (WT) embryos as a dark line devoid of filamentous actin (Figure 7A, white arrow). In *wi390/lamγ1* mutant embryos, some muscle fibers inappropriately cross the MTJ and are approximately twice as long as their counterparts that did not cross the boundary (Figure 7 A1 red arrowhead). These cells are multinucleate (data not shown), indicating that boundary capture is not required for fusion. The crossing of a boundary by a few muscle fibers results in an asymmetrical myotome: some of the myotome has longer fibers while the majority of fibers are an appropriate length (Figure 7 A1). Fast fibers in *gup/lamβ1* mutants also cross MTJ boundaries (Figure 7 A2 red arrowhead). At 48 hpf, some boundaries were crossed within every *laminin*-deficient embryo examined. Generally, 16–24% of boundaries were crossed (average % of boundaries crossed: WT, 0%, n>100; *gup/lamβ1*, 20% crossed, n = 12 embryos, 3 experiments; *wi390/lamγ1*, 22% crossed, n = 9 embryos, 1 experiment; *lamβ1* MO, 24% crossed, n = 26 embryos, 5 experiments; *lamγ1* MO, 16% crossed, n = 18 embryos, 3 experiments).

### Boundary Capture as a Cell Autonomous Phenomenon

Our data show that *lamβ1* and *γ1* play a role in boundary capture of elongating muscle fibers, but the mechanism of capture is not yet known. A dense network of polymerized laminin may function as a physical barrier that stops elongating muscle fibers. Interestingly, however, laminin polymerization can trigger changes in the organization of the matrix, ECM receptors and cytoskeletal components [36]. Cell-autonomous changes in cytoskeletal organization upon laminin binding provide an alternate hypothesis: that signaling that results from laminin binding may mediate boundary capture in a cell-autonomous fashion. We hypothesized that WT cells transplanted in laminin-deficient embryos might be able to secrete small amounts of laminin that would facilitate their capture and reduce the likelihood of elongating through the boundary. To test this, cells from dextran-injected control embryos were transplanted into *lamγ1* morphant hosts. Control cells were less likely than *lamγ1* morphant cells to cross the boundary (Figure 7C, D). Only 6 percent of control cells crossed boundaries (19/311 cells) whereas 25% of morphant cells crossed boundaries (407/1631 cells). Control cells undergo boundary capture even when adjacent to *lamγ1* morphant cells crossing boundaries (Figure 7 B1, B2 note that the red control cell, white arrowhead respects the boundary, but adjacent morphant cells cross the boundary, red arrowhead). These data not only provide the first evidence that laminin plays a role in ceasing initial myofiber elongation, but the cell autonomous rescue of boundary integrity by WT cells suggests that boundary capture is mediated at the single cell level.

## Discussion

A mechanistic understanding of the cellular basis of muscle cell elongation and tendon attachment is critical to elucidate underlying molecular mechanisms that mediate morphogenesis. We show here the first quantitative analysis of *individual* fast muscle cell elongation in a living vertebrate embryo. Three broad phases of morphogenesis underlie the transition from a somite comprised of short muscle precursor cells to a myotome comprised of elongated muscle fibers. First, short muscle precursor cells exhibit dynamic protrusive activity, but do not undergo large-scale shape changes. The second phase, intercalation/elongation, occurs via a repetitive two-step process of protrusion extension and filling and requires *lamβ1* and *γ1* to proceed efficiently. The third phase encompasses boundary capture as well as shape changes that generate a more regularly shaped myotube. Although myotubes do form in *laminin*-deficient embryos, they are significantly less organized than in wild-type embryos. We find that both *lamβ1* and *γ1* are required for boundary capture and thus provide the first molecular insight into boundary capture at the MTJ. Taken together, these data indicate that muscle morphogenesis is spatiotemporally complex and involves interactions between muscle fibers and the basement membrane during elongation and attachment to the MTJ. It is not yet known if there is some conservation between morphogenetic mechanisms underlying early morphogenesis between vertebrates. Given recent data indicating that the zebrafish somite has a dermomyotome and is thus more homologous to amniotes as previously thought [37]–[39], it is tempting to speculate that the morphogenetic mechanisms described here may apply to higher vertebrates as well.

### Three Phases of Early Fast Muscle Morphogenesis

Both qualitative and quantitative assessments of early muscle development are critical to facilitate identification of molecular mechanisms that underlie morphogenesis. We find that the three phases of early fast muscle morphogenesis are qualitatively and quantitatively different. These stages are short muscle precursor cells, elongating muscle cells and myotube formation. Short muscle precursor cells have a low filament index and extend and retract short (<2 µm) protrusions in all directions. Elongating fast muscle cells extend long protrusions along the axis of elongation and have a higher filament index. Long muscle cells forming myotubes have an even higher filament index indicating yet a further departure from a circular shape. Thus, we provide a novel paradigm whereby morphometric analysis can distinguish different phases of early muscle development.

### Mathematical Modeling and Time-Lapse Analysis Indicate that a Repetitive Two-Step Mechanism Underlies Fast Muscle Cell Elongation

**Figure 8:**
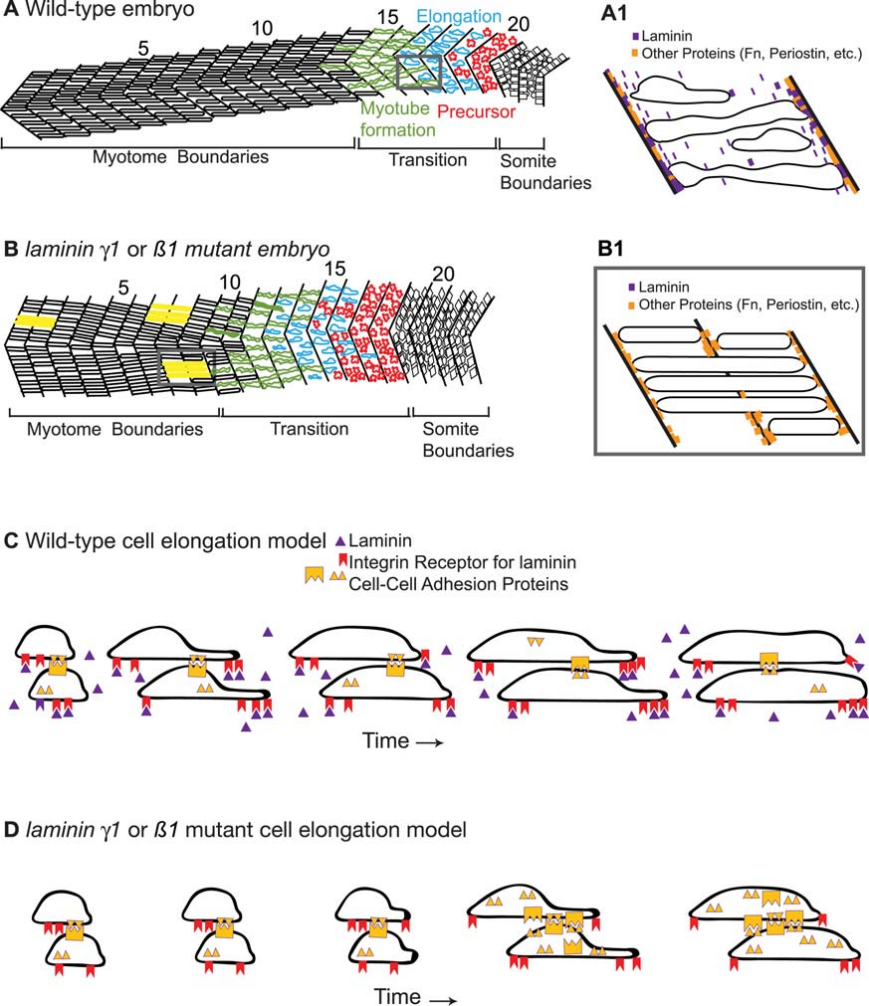
Model of Muscle Morphogenesis in WT and *laminin* Mutant Embryos. A–A1) Cartoon of WT embryo showing the three phases of muscle elongation. In the oldest/most anterior somites, myotubes have formed and are attached to the MTJs. The transition region contains cells intercalating by extending protrusions that are subsequently filled. Muscle precursor cells exhibit protrusive activity in all directions. A1: Magnification of a somite in panel A showing proteins concentrated at the MTJ and boundary capture of recently elongated cells. B–B1) Cartoon of *laminin* mutant embryo at the same age as WT embryo in panel A showing the same three phases of muscle morphogenesis, but with a developmental delay. Cells in yellow are aberrantly long and have invaded into neighboring myotomes. B1: Magnification of two somites in panel B that depicts a model of how boundary crossing could occur in *laminin* mutant embryos. If laminin is absent, there may be randomly spaced locations at the MTJ devoid of proteins that function in boundary capture. Elongating muscle cells would invade the MTJ at these locations. C) Cartoon model showing the two-step mechanism of elongating. We show that adhesion to the matrix is required for normal elongation and hypothesize that cells also utilize cell-cell adhesion to generate traction forces needed for protrusion extension and filling. D) Model accounting for developmental delay in muscle morphogenesis that occurs in *laminin* mutants. Cartoon depicting a *laminin* mutant cell undergoing two-step elongation via protrusion extension and filling. Lack of the cell-matrix adhesion protein laminin results in less traction and therefore slower extension and/or filling.

It is not known how the first fast-twitch muscle cells elongate during vertebrate development. We utilized an experimental approach to distinguish between potential scenarios (Figure 2A). C2C12 myoblasts in culture elongate prior to differentiation and fuse to generate a multinucleate myotube [10] that we termed the elliptical growth scenario. The first muscle cells to elongate in grasshopper embryos (muscle pioneers) exhibit a morphology similar to that of pathfinding neurons [11], we have called this the branching scenario. During *Drosophila* embryogenesis, muscle cells elongate via fusion [12],[13] and zebrafish homologues of genes required for muscle cell fusion in *Drosophila* are also required for normal muscle development in zebrafish [14],[15]. It has also been proposed that zebrafish muscle cell elongation may be similar to notochord/neural plate cell intercalation [24], represented by the protrusion scenario. Time-lapse analysis indicates that elongating cells extend local protrusions along their long axis (Figure 8C, D). Protrusions are extended in the direction of elongation and between other cells. Protrusions then thicken, resulting in elongation of the cell. Repetition of protrusion extension/thickening results in an elongated muscle cell. Mathematical modeling of expected changes in area and perimeter supports the protrusion model of morphogenesis. Thus, we show that a novel two-step mechanism underlies elongation of the first fast muscle fibers in a vertebrate model system, the zebrafish.

### Muscle Cell Fusion

Muscle development is perhaps best understood in *Drosophila*, where muscle morphogenesis is accomplished via fusion of founder cells (FCs) with fusion competent myoblasts (FCMs) [13]. Recent 3-D imaging has demonstrated that there are two phases of fusion and suggests that the spatial relationship of FCs and FCMs influences the frequency of fusion events [12]. Exciting recent studies using zebrafish suggest that molecular events underlying muscle cell fusion in vertebrates may be at least partially conserved [14],[15],[40]. In the future it will be important to understand the cellular basis of fusion as well. In this regard, we show that elongating/recently elongated muscle cells possess complex 3-D shapes. Thus, a comprehensive analysis of cell behaviors underlying muscle cell fusion during zebrafish development will require development of multiple markers that label entire muscle cells such that fusion can unambiguously be analyzed. Genetic mosaic approaches such as those used previously [41] will facilitate analysis of both the timing of fusion as well as identifying what cells fuse.

### Attachment to Laminin Is Necessary for Timely Fast Muscle Cell Elongation

We show that *lamβ1* and *γ1* are required for efficient fast muscle cell elongation and proper organization. Application of the 2D WTMM method indicates that even in early stages of muscle development where organizational differences are not visually obvious, anisotropic signatures reveal unequivocally the morphological discrepancies between *laminin*-deficient and control embryos. This emphasizes the strength of the 2D WTMM method. This novel use of the 2D WTMM method will give researchers an invaluable tool to rigorously and quantitatively distinguish subtle differences in cellular morphology and organization.

We do not know why fast muscle cell elongation is delayed in *lamβ1* and *γ1*-deficient embryos. Elongation may be delayed because fast cells are less organized than in controls. It is also possible that fast cells in *lamβ1* and *γ1* mutant/morphant embryos do not elongate efficiently because slow muscle cells do not migrate efficiently. Although WT slow fibers can rescue elongation in mutant embryos that do not have slow muscle fibers [28], it is unknown if disrupted slow muscle migration and/or morphology may delay fast muscle cell elongation.

A third model is that adhesion to laminin may play a role in generation of traction forces that allow muscle cells to elongate. Muscle cells extend protrusions as they elongate and these protrusions likely attach to other cells or the ECM. Attachment would provide a mechanism for cells to stabilize an extended protrusion and continue elongation. Interestingly, adhesion to laminin via the Integrin α7β1 receptor promotes migration of C2C12 and MM14 cells in culture [42]. Elongating fast muscle cells in zebrafish do not migrate per se, but future studies will address whether adhesion to laminin during fast muscle cell elongation in zebrafish promotes efficient protrusion extension and thickening. These studies would be facilitated by identification of the relevant laminin receptor (there are multiple laminin receptors) such that genetic mosaic analysis could readily be used.

Fast muscle cells do belatedly elongate in the absence of laminin. It is possible, even likely, that elongating muscle cells may utilize different modes of adhesion to the substrate and/or other cells. Thus, if one mode of adhesion is disrupted, muscle cell elongation would be delayed, but not entirely inhibited. Our results indicating that muscle cell elongation is delayed, rather than inhibited, are similar to the finding that myofiber formation is delayed, but recovers in mouse knockouts of the cell-cell adhesion protein CDO [43]. Taken together, these results suggest that muscle cells elongate by extension of protrusions that adhere both to other cells and the ECM. If one mode of adhesion is disrupted, cells are delayed in their elongation, but utilize the alternative mode of adhesion to eventually elongate (Figure 8C, D).

### Laminin Participates in Boundary Capture of Elongating Muscle Cells

One fundamental process during embryonic development is boundary formation. Some of the first work describing boundary formation was done by Jacobson and colleagues [44]–[46], where they showed that cells that reach the notoplate/neural plate boundary remain on the boundary permanently in both axolotl and newt embryos. This phenomenon was referred to as trapping. Keller and colleagues have since expanded upon this model and termed it boundary capture [47]. Recent work demonstrated that *laminin* plays a critical role in boundary capture during notochord morphogenesis in the ascidian *Ciona savignyi* [48]. We have previously demonstrated that the MTJ captures elongating muscle fibers, but it was not known what ECM components were relevant [24]. It was also not known if the cessation of muscle fiber elongation is cell autonomous or mediated by community effects. Here we show that laminin is one component of the MTJ that stops elongating fibers. This result, combined with the work of Veeman et al., suggests that roles for laminin in boundary capture may be conserved, at least within chordates. We also show that wild-type cells in *lamγ1* morphant embryos have a reduced ability to cross the MTJ. The fact that wild-type cells are less able to cross the MTJ, but do not rescue their *lamγ1*-deficient neighbors, suggests that boundary capture is a cell autonomous process. These data also suggest that MTJ breakdown in *lamβ1*and *γ1*-deficient embryos is a local event caused by the failure of elongating muscle fibers to stop when they reach the MTJ. We do not currently know why 75% of elongating muscle cells in *lamβ1* and *γ1*-deficient embryos do stop elongating, but 25% do not. We hypothesize that the MTJ boundary is not homogenous. In this scenario, the absence of laminin would leave “holes” in the MTJ and muscle cells would elongate through these holes (Figure 8B, B1). Future experiments will be directed towards identifying additional molecular cues involved in boundary capture.

## Methods

### Zebrafish Husbandry

Zebrafish embryos were obtained from natural spawnings of adult fish kept at 28.5°C on a 16 h light/8 h dark cycle and were staged according to [49].

### Immunocytochemistry

F59 was utilized to visualize slow fibers as previously described [25],[50]. Alexa Fluor 488 and 546 phalloidin and Sytox green were obtained from Molecular Probes. We used the H2A∶GFP transgenic line of zebrafish to visualize nuclei [51]. A “scatter” label of cells filled with fluoro-ruby dextrans (Molecular Probes) was obtained by microinjecting embryos at the 512–1000 cell stage with dextrans into the yolk cell close to the margin.

Antibodies used were: mouse monoclonal anti-myosin (F59) (Devoto, et al. 1996, generous gift of Frank Stockdale) 1∶10, mouse monoclonal anti-β-catenin (Sigma) 1∶500 and Alexa-Fluor 488, 546 and 633 conjugated goat anti-mouse and goat anti-rabbit secondary antibodies (Invitrogen) 1∶200.

### Imaging

Images were acquired using a Leica SP2 confocal microscope and a Zeiss ApoTome running on a Zeiss Axio Imager Z1. All mathematical analyses were done on images acquired on the Apotome using a 20× lens, NA 0.8, yielding a resolution of 1.5 pixels / µm. Images were linearly processed in Adobe Photoshop and collated in Adobe Illustrator.

### Morpholinos

Morpholino-modified antisense oligonucleotides (MOs) were synthesized by Gene-Tools, LCC. The morpholinos used were previously described and recapitulate the mutant phenotypes [31].

### Time-Lapse Analysis

Embryos were vitally stained and imaged with the fluorescent, lipophilic dye BODIPY-Ceramide (Molecular Probes, Eugene, OR) using the procedures outlined by [52],[53]. Time-lapse recordings were made using a scanning laser confocal microscope (Leica SP2, Heidelburg, Germany). Time-lapse analysis with transplanted dextran-filled cells was performed utilizing the Zeiss ApoTome.

### Morphometrics

To measure properties of dextran-filled cells, the z-series of the cell was projected such as to visualize the cell three-dimensionally. Cells were then segmented with ImageJ and the perimeter, area and major axis were measured. The major axis as determined by ImageJ is the longest length of the best fitting ellipse. The filament index was also calculated [54]:
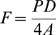
where *P*, *D* and *A* are the perimeter, diameter and area respectively. For this study, the diameter was taken to be equal to the major axis. Note that a circle has a filament index *F* = 1 and an object having a value of *F* larger than 1 quantifies its departure from a circular shape. A two sample T-test was performed using SYSTAT. * denotes p<0.05 and ** denotes p<0.01.

### Mathematical Modeling

In order to quantitatively characterize the morphology of muscle fiber growth, two geometrical models were developed. For simplicity, both the elliptical and protrusion models start with a unit circle (with radius = 1). For both models, when the cell is growing we assume that it does so only in the major direction and that the semi-minor axis stays constant, and for mathematical simplicity (and without any loss of generality), is equal to 1. Therefore, we have
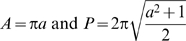
The growth ratios for the area and perimeter of both models can be defined analytically. For the elliptical model, the growth ratio for the area at time *t*, *Â*_elliptical_(*t*), is equal to the major axis at time *t*, which grows continuously:
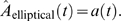
The perimeter growth ratio at time *t*, *Pˆ*_elliptical_(*t*), is given by
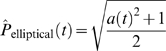
For the protrusion model, the cell grows in a two-step manner, expanding a thin protrusion of relatively small area Δ*A* and perimeter Δ*P* at time *t*, and then filling the area until the cell becomes an ellipse at time *t*+1. Therefore, the growth ratio for the area of the protrusion model will depend on whether it is growing a protrusion or filling that protrusion. For simplicity, we assume that the cell is growing a protrusion if *t* is even and it is filling the area opened by protrusion when *t* is odd:
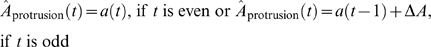
Similarly for the perimeter of the protrusion model cell:
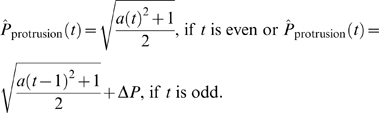
Since Δ*A* is relatively small with respect to the area of the whole cell, the extension of a protrusion will not significantly increase the area of the cell. Conversely, since Δ*P* is relatively large with respect to the perimeter of the whole cell, the perimeter of the cell will significantly increase. The evolution of the area and perimeter as a function of time for both models is shown in Figure 3A, A1.

### Characterizing Anisotropy with the 2D WTMM Method

#### Preparation of images

Embryos were stained with β-catenin to outline all cells and fixed at 20 hpf. This stage is ideal because anterior, older somites have formed muscle but posterior somites have not. Thus, all the phases of muscle morphogenesis are represented within single embryos. Images representing all phases were obtained utilizing a Zeiss Axioimager equipped with an Apotome as mentioned above. Each image and focal plane was evaluated and the phase (short precursor, etc.) determined. The phase that was identified represented most cells within a focal plane. Because muscle cell elongation is an exceedingly dynamic process, cells within different focal planes within the same z-series are sometimes at different phases of elongation. Each image was then cropped into 256×256 pixel sub-images. This cropping is necessary to eliminate other tissues in the embryos such as residual yolk platelets and neural tissue. Cropping was also necessary because fast muscle cells in the dorsal and ventral halves of somites angle slightly towards the middle. We thus flipped all ventrally derived panels so that the WTMMM vector angles for dorsal and ventral halves would not cancel each other out within a single sub-image. At least 10 images for at least 5 different embryos were analyzed for each phase.

The 2D WTMM method is a multifractal image analysis formalism introduced in [34], where the different dilations of the analyzing wavelet reveal quantitative roughness information at every length scale considered. By considering two wavelets that are, respectively, the partial derivatives with respect to *x* and *y* of a 2D smoothing Gaussian function, the Wavelet Transform is thus the gradient vector of the analyzed image smoothed by dilated versions of the Gaussian filter. A very efficient way to perform point-wise regularity analysis is to use the Wavelet Transform Modulus Maxima (WTMM) [55],[56]. At a given scale *a*, the WTMM are defined by the positions where the Wavelet Transform Modulus is locally maximum in the direction *A* of the gradient vector. When analyzing rough surfaces, these WTMM lie on connected chains called *maxima chains* [34], as shown in Figure 5, green lines. One only needs to record the position of the local maxima of the gradient along the maxima chains together with the angle *A* at the corresponding locations. At each scale *a*, the wavelet analysis thus reduces to store those WTMM maxima (WTMMM) only (red dots in Figure 5). They indicate locally the direction where the signal has the sharpest variation.

An image having an anisotropic signature means that the intensity variation in the image will differ according to the direction considered. Such images having an anisotropic signature can be easily characterized from the directional information provided by the continuous 2D Wavelet Transform [35]. This is done by considering, at all size scales *a*, the probability density functions (pdfs), *P_a_*(*A*), of the angles, *A*, associated to each WTMMM vector. A flat pdf indicates unprivileged random directions of sharpest intensity variation (i.e. isotropy), while any departure from a flat distribution is interpreted as the signature of anisotropy. For the present study, a strong anisotropic signature is interpreted as a strongly structured cell lattice.

### Anisotropy Factor

In order to obtain quantitative information from the angle pdfs *P_a_*(*A*), they are compared to a theoretical flat distribution representing an ideal isotropic signature (see Figure 5F). The *anisotropy factor*, *F_a_*, defined for each value of the scale parameter *a*, is given by the area between the curve corresponding to the observed pdfs and a flat distribution:
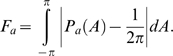



Therefore *F_a_* has been defined in such a way that a theoretically isotropic surface will have a value of *F_a_* = 0, while any value greater than 0 quantifies a departure from isotropy.

### Construction of Simulated Isotropic Surfaces for Calibration Purposes

Following the standard procedures presented in [34],[35], fractional Brownian motion (fBm) isotropic surfaces were generated. Two-dimensional fBm's are processes with stationary zero-mean Gaussian increments that are statistically invariant under isotropic dilations. They are therefore expected to reproduce quite faithfully the isotropic scaling invariance properties.

### Genetic Mosaic Analysis

WT embryos were injected with 10,000 MW dextrans (Molecular Probes). Cells were removed at the sphere stage and placed into hosts that had been injected with *lamγ1* MOs. Hosts were grown up until the appropriate stage, stained with phalloidin and the number of transplanted control cells that crossed MTJ boundaries was compared with the number of *lamγ1* morphant cells that crossed MTJ boundaries.

## Supporting Information

Figure S1 Somite Boundary Shape, Slow Muscle Migration and Fast Muscle Elongation are Disrupted in lamininβ1 and γ1-deficient Embryos. All panels are ApoTome images at the 18 somite stage. Side views, anterior left, dorsal top, except panels numbered 4 that are transverse views, lateral left, medial right. Panels 2–4 are higher magnification views of the embryos shown in panels numbered 1. Panels numbered 1 and 2 are single focal planes from a Z-series and show phalloidin staining that outlines all cells. Panels numbered 3 and 4 are projections of the entire Z-series of panels numbered 2. In these panels, F59 expression denotes slow-twitch muscle fibers. All panels (A1–C1, A2–C2) are from approximately the same anterior-posterior and medial-lateral position in control and morphant embryos. A1–C1) WT control embryos contain robust, chevron shaped boundaries. *lamβ1* and *lamγ1* morphants have rounder, flatter shaped boundaries. Note that intial somite boundaries, albeit less chevron-shaped, do form in *lamβ1* and *lamγ1* morphant embryos. A2–C2) Whereas fast muscle cells are elongating in control embryos (A2, white arrow), fast-twitch muscle cell elongation is disrupted in both *lamβ1* (B2, white arrowhead) and *lamγ1* (C2, white arrowhead) morphant embryos but some elongation does occur (white arrows). A3–C3/A4–C4) Myosin organization in slow-twitch muscle fibers is disrupted in *lamβ1* and *lamγ1* morphant embryos. In control embryos, the projected (panels numbered 3) and rotated transverse views (panels numbered 4) show organized slow-twitch fibers that have migrated laterally (muscle pioneers: red asterisk). Slow-twitch fiber organization, spacing, and migration, are disrupted in *lamβ1* and *lamγ1* morphant embryos.
(7.8 MB TIF)

Movie S1 Time-lapse confocal microscopy using BODIPY-ceramide to outline cell borders suggests that cells intercalate during elongation. Side views, anterior left. Colored cells were tracked in Image J and pseudocolored in Adobe Photoshop. The purple cell initiates elongation as does the orange cell. The blue cell elongates through time, extending a thin protrusion between the orange and green cells eventually reaching the anterior boundary. The long but irregularly shaped green cell becomes a rod shaped myotube through time.
(2.0 MB MOV)

Movie S2 Time-lapse analysis of three-dimensional projections from ApoTome micrographs shows the three phases of morphogenesis. Short dextran-filled cells exhibit protrusive activity as they begin elongating. Elongating cells initially extend long protrusions that subsequently grow, resulting in the elongation of the cell. Green arrowheads denote extensions. Cells in the myotube formation phase are initially irregularly shaped but become rod shaped myotubes through time.
(1.0 MB MOV)

Movie S3 Three-dimensional shapes of fixed cells in different phases of morphogenesis. Part 1: A lateral-medial Z-series of a long yet irregularly shaped dextran-filled cell with one nucleus. Part 2: Rotation of a three-dimensional projection of a partially elongated dextran-filled cell in a fixed embryo showing an extension. Part 3: Rotation of a three-dimensional projection of myotube in a fixed embryo.
(2.7 MB MOV)

Movie S4 Time-lapse analysis of live cells.
(1.5 MB MOV)

## References

[1] Hollway G, Currie P (2005). Vertebrate myotome development.. Birth Defects Res C Embryo Today.

[2] Hollway GE, Currie PD (2003). Myotome meanderings. Cellular morphogenesis and the making of muscle.. EMBO Rep.

[3] Buckingham M, Bajard L, Chang T, Daubas P, Hadchouel J (2003). The formation of skeletal muscle: from somite to limb.. J Anat.

[4] Long JH, Adcock B, Root RG (2002). Force transmission via axial tendons in undulating fish: a dynamic analysis.. Comp Biochem Physiol A Mol Integr Physiol.

[5] Kahane N, Cinnamon Y, Kalcheim C (1998). The cellular mechanism by which the dermomyotome contributes to the second wave of myotome development.. Development.

[6] Denetclaw WF, Ordahl CP (2000). The growth of the dermomyotome and formation of early myotome lineages in thoracolumbar somites of chicken embryos.. Development.

[7] Ben-Yair R, Kalcheim C (2005). Lineage analysis of the avian dermomyotome sheet reveals the existence of single cells with both dermal and muscle progenitor fates.. Development.

[8] Gros J, Scaal M, Marcelle C (2004). A two-step mechanism for myotome formation in chick.. Dev Cell.

[9] Waterman RE (1969). Development of the lateral musculature in the teleost, Brachydanio rerio: a fine structural study.. Am J Anat.

[10] Ohtake Y, Tojo H, Seiki M (2006). Multifunctional roles of MT1-MMP in myofiber formation and morphostatic maintenance of skeletal muscle.. J Cell Sci.

[11] Ho RK, Ball EE, Goodman CS (1983). Muscle pioneers: large mesodermal cells that erect a scaffold for developing muscles and motoneurones in grasshopper embryos.. Nature.

[12] Beckett K, Baylies MK (2007). 3D analysis of founder cell and fusion competent myoblast arrangements outlines a new model of myoblast fusion.. Dev Biol.

[13] Chen EH, Olson EN (2004). Towards a molecular pathway for myoblast fusion in Drosophila.. Trends Cell Biol.

[14] Moore CA, Parkin CA, Bidet Y, Ingham PW (2007). A role for the Myoblast city homologues Dock1 and Dock5 and the adaptor proteins Crk and Crk-like in zebrafish myoblast fusion.. Development.

[15] Srinivas BP, Woo J, Leong WY, Roy S (2007). A conserved molecular pathway mediates myoblast fusion in insects and vertebrates.. Nat Genet.

[16] LeBleu VS, Macdonald B, Kalluri R (2007). Structure and function of basement membranes.. Exp Biol Med (Maywood).

[17] Hoffman EP, Brown RH, Kunkel LM (1987). Dystrophin: the protein product of the Duchenne muscular dystrophy locus.. Cell.

[18] Bonnemann CG, Modi R, Noguchi S, Mizuno Y, Yoshida M (1995). Beta-sarcoglycan (A3b) mutations cause autosomal recessive muscular dystrophy with loss of the sarcoglycan complex.. Nat Genet.

[19] Noguchi S, McNally EM, Ben Othmane K, Hagiwara Y, Mizuno Y (1995). Mutations in the dystrophin-associated protein gamma-sarcoglycan in chromosome 13 muscular dystrophy.. Science.

[20] Nigro V, de Sa Moreira E, Piluso G, Vainzof M, Belsito A (1996). Autosomal recessive limb-girdle muscular dystrophy, LGMD2F, is caused by a mutation in the delta-sarcoglycan gene.. Nat Genet.

[21] Hall TE, Bryson-Richardson RJ, Berger S, Jacoby AS, Cole NJ (2007). The zebrafish candyfloss mutant implicates extracellular matrix adhesion failure in laminin {alpha}2-deficient congenital muscular dystrophy.. Proc Natl Acad Sci U S A.

[22] Bajanca F, Luz M, Raymond K, Martins GG, Sonnenberg A (2006). Integrin alpha6beta1-laminin interactions regulate early myotome formation in the mouse embryo.. Development.

[23] Nishiuchi R, Murayama O, Fujiwara H, Gu J, Kawakami T (2003). Characterization of the ligand-binding specificities of integrin alpha3beta1 and alpha6beta1 using a panel of purified laminin isoforms containing distinct alpha chains.. J Biochem.

[24] Henry CA, McNulty IM, Durst WA, Munchel SE, Amacher SL (2005). Interactions between muscle fibers and segment boundaries in zebrafish.. Dev Biol.

[25] Devoto SH, Melancon E, Eisen JS, Westerfield M (1996). Identification of separate slow and fast muscle precursor cells in vivo, prior to somite formation.. Development.

[26] Blagden CS, Currie PD, Ingham PW, Hughes SM (1997). Notochord induction of zebrafish slow muscle mediated by Sonic hedgehog.. Genes Dev.

[27] Cortes F, Daggett D, Bryson-Richardson RJ, Neyt C, Maule J (2003). Cadherin-mediated differential cell adhesion controls slow muscle cell migration in the developing zebrafish myotome.. Dev Cell.

[28] Henry CA, Amacher SL (2004). Zebrafish slow muscle cell migration induces a wave of fast muscle morphogenesis.. Dev Cell.

[29] Kudo H, Amizuka N, Araki K, Inohaya K, Kudo A (2004). Zebrafish periostin is required for the adhesion of muscle fiber bundles to the myoseptum and for the differentiation of muscle fibers.. Dev Biol.

[30] Crawford BD, Henry CA, Clason TA, Becker AL, Hille MB (2003). Activity and distribution of paxillin, focal adhesion kinase, and cadherin indicate cooperative roles during zebrafish morphogenesis.. Mol Biol Cell.

[31] Parsons MJ, Pollard SM, Saude L, Feldman B, Coutinho P (2002). Zebrafish mutants identify an essential role for laminins in notochord formation.. Development.

[32] Julich D, Geisler R, Holley SA (2005). Integrinalpha5 and delta/notch signaling have complementary spatiotemporal requirements during zebrafish somitogenesis.. Dev Cell.

[33] Koshida S, Kishimoto Y, Ustumi H, Shimizu T, Furutani-Seiki M (2005). Integrinalpha5-dependent fibronectin accumulation for maintenance of somite boundaries in zebrafish embryos.. Dev Cell.

[34] Arneodo A, Decoster N, Roux S (2000). A wavelet-based method for multifractal image analysis. I. Methodology and test applications on isotropic and anisotropic random rough surfaces.. European Journal of Physics B.

[35] Khalil A, Joncas G, Nekka F, Kestener P, Arneodo A (2006). Morphological analysis of HI features. II. Wavelet-based multifractal formalism.. Astrophysical Journal Supplement Series.

[36] Colognato H, Winkelmann DA, Yurchenco PD (1999). Laminin polymerization induces a receptor-cytoskeleton network.. J Cell Biol.

[37] Stellabotte F, Devoto SH (2007). The teleost dermomyotome.. Dev Dyn.

[38] Hammond CL, Hinits Y, Osborn DP, Minchin JE, Tettamanti G (2007). Signals and myogenic regulatory factors restrict pax3 and pax7 expression to dermomyotome-like tissue in zebrafish.. Dev Biol.

[39] Devoto SH, Stoiber W, Hammond CL, Steinbacher P, Haslett JR (2006). Generality of vertebrate developmental patterns: evidence for a dermomyotome in fish.. Evol Dev.

[40] Krauss RS (2007). Evolutionary conservation in myoblast fusion.. Nat Genet.

[41] Roy S, Wolff C, Ingham PW (2001). The u-boot mutation identifies a Hedgehog-regulated myogenic switch for fiber-type diversification in the zebrafish embryo.. Genes Dev.

[42] Yao CC, Ziober BL, Sutherland AE, Mendrick DL, Kramer RH (1996). Laminins promote the locomotion of skeletal myoblasts via the alpha 7 integrin receptor.. J Cell Sci.

[43] Cole F, Zhang W, Geyra A, Kang JS, Krauss RS (2004). Positive regulation of myogenic bHLH factors and skeletal muscle development by the cell surface receptor CDO.. Dev Cell.

[44] Jacobson AG, Moury JD (1995). Tissue boundaries and cell behavior during neurulation.. Dev Biol.

[45] Jacobson AG, Oster GF, Odell GM, Cheng LY (1986). Neurulation and the cortical tractor model for epithelial folding.. J Embryol Exp Morphol.

[46] Moury JD, Jacobson AG (1989). Neural fold formation at newly created boundaries between neural plate and epidermis in the axolotl.. Dev Biol.

[47] Keller R, Davidson L, Edlund A, Elul T, Ezin M (2000). Mechanisms of convergence and extension by cell intercalation.. Philos Trans R Soc Lond B Biol Sci.

[48] Veeman MT, Nakatani Y, Hendrickson C, Ericson V, Lin C (2008). Chongmague reveals an essential role for laminin-mediated boundary formation in chordate convergence and extension movements.. Development.

[49] Kimmel CB, Ballard WW, Kimmel SR, Ullmann B, Schilling TF (1995). Stages of embryonic development of the zebrafish.. Dev Dyn.

[50] Crow MT, Stockdale FE (1986). Myosin expression and specialization among the earliest muscle fibers of the developing avian limb.. Dev Biol.

[51] Pauls S, Geldmacher-Voss B, Campos-Ortega JA (2001). A zebrafish histone variant H2A.F/Z and a transgenic H2A.F/Z:GFP fusion protein for in vivo studies of embryonic development.. Dev Genes Evol.

[52] Cooper MS, D'Amico LA, Henry CA (1999). Analyzing morphogenetic cell behaviors in vitally stained zebrafish embryos.. Methods Mol Biol.

[53] Cooper MS, D'Amico LA, Henry CA (1999). Confocal microscopic analysis of morphogenetic movements.. Methods Cell Biol.

[54] Khalil A, Grant JL, Caddle LB, Atzema E, Mills KD (2007). Chromosome territories have a highly nonspherical morphology and nonrandom positioning.. Chromosome Res.

[55] Mallat S, Hwang WL (1992). IEEE Trans on Information Theory.

[56] Mallat S, Zhong S (1992). IEEE Trans on Patern Analysis and Machine Intelligence.

