# Supplementary figures and images for: Time-Lapse Analysis and Mathematical Characterization Elucidate Novel Mechanisms Underlying Muscle Morphogenesis
Source: PLoS Genet. 2008 Oct 3;4(10):e1000219. doi: 10.1371/journal.pgen.1000219 (PMC2543113; doi:10.1371/journal.pgen.1000219)

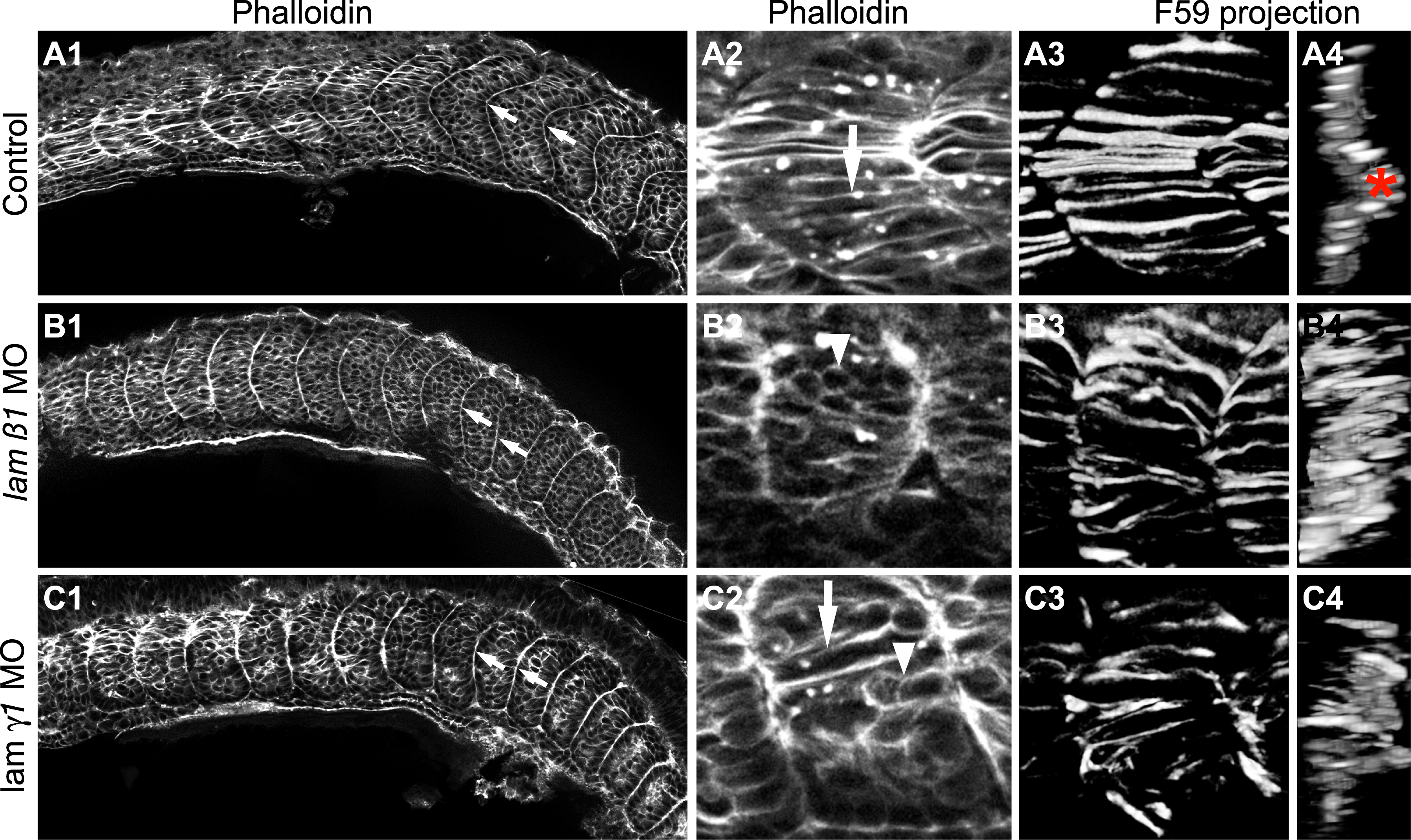

Supplement: Figure S1 — Somite Boundary Shape, Slow Muscle Migration and Fast Muscle Elongation are Disrupted in lamininβ1 and γ1-deficient Embryos. All panels are ApoTome images at the 18 somite stage. Side views, anterior left, dorsal top, except panels numbered 4 that are transverse views, lateral left, medial right. Panels 2–4 are higher magnification views of the embryos shown in panels numbered 1. Panels numbered 1 and 2 are single focal planes from a Z-series and show phalloidin staining that outlines all cells. Panels numbered 3 and 4 are projections of the entire Z-series of panels numbered 2. In these panels, F59 expression denotes slow-twitch muscle fibers. All panels (A1–C1, A2–C2) are from approximately the same anterior-posterior and medial-lateral position in control and morphant embryos. A1–C1) WT control embryos contain robust, chevron shaped boundaries. lamβ1 and lamγ1 morphants have rounder, flatter shaped boundaries. Note that intial somite boundaries, albeit less chevron-shaped, do form in lamβ1 and lamγ1 morphant embryos. A2–C2) Whereas fast muscle cells are elongating in control embryos (A2, white arrow), fast-twitch muscle cell elongation is disrupted in both lamβ1 (B2, white arrowhead) and lamγ1 (C2, white arrowhead) morphant embryos but some elongation does occur (white arrows). A3–C3/A4–C4) Myosin organization in slow-twitch muscle fibers is disrupted in lamβ1 and lamγ1 morphant embryos. In control embryos, the projected (panels numbered 3) and rotated transverse views (panels numbered 4) show organized slow-twitch fibers that have migrated laterally (muscle pioneers: red asterisk). Slow-twitch fiber organization, spacing, and migration, are disrupted in lamβ1 and lamγ1 morphant embryos. (7.8 MB TIF) [file pgen.1000219.s001.tif]
